# Recent History and Geography of Virtual Water Trade

**DOI:** 10.1371/journal.pone.0055825

**Published:** 2013-02-15

**Authors:** Joel A. Carr, Paolo D’Odorico, Francesco Laio, Luca Ridolfi

**Affiliations:** 1 Department of Environmental Sciences, University of Virginia, Charlottesville, Virginia, United States of America; 2 Department of Environmental, Land and Infrastructure Engineering, Politecnico di Torino, Turin, Italy; Cinvestav-Merida, Mexico

## Abstract

The global trade of goods is associated with a virtual transfer of the water required for their production. The way changes in trade affect the virtual redistribution of freshwater resources has been recently documented through the analysis of the virtual water network. It is, however, unclear how these changes are contributed by different types of products and regions of the world. Here we show how the global patterns of virtual water transport are contributed by the trade of different commodity types, including plant, animal, luxury (e.g., coffee, tea, and alcohol), and other products. Major contributors to the virtual water network exhibit different trade patterns with regard to these commodity types. The net importers rely on the supply of virtual water from a small percentage of the global population. However, discrepancies exist among the different commodity networks. While the total virtual water flux through the network has increased between 1986 and 2010, the proportions associated with the four commodity groups have remained relatively stable. However, some of the major players have shown significant changes in the virtual water imports and exports associated with those commodity groups. For instance, China has switched from being a net exporter of virtual water associated with other products (non-edible plant and animal products typically used for manufacturing) to being the largest importer, accounting for 31% of the total water virtually transported with these products. Conversely, in the case of The United states of America, the commodity proportions have remained overall unchanged throughout the study period: the virtual water exports from The United States of America are dominated by plant products, whereas the imports are comprised mainly of animal and luxury products.

## Introduction

Food production requires adequate climate, soils, and water availability. Recently, there has been renewed focus on various issues surrounding water resources including: water for sanitation and drinking [1], [2], water as a source of conflict [3], [4], and water use in the growth of food [5]. In particular, with an ever increasing global population, the ability to maintain adequate food supplies with limited water resources has become a pressing concern [6], [7]. When a society has no access to favorable conditions to produce all the food it needs, its demand can be met through importations from other regions. Food trade allows some populations to thrive in water deficit regions by avoiding or mitigating water stress conditions. It has been noted [8], [9] that the trade of agricultural and industrial commodities is associated with a virtual transfer of the water resources used for the production of these goods [10]. Global trade virtually transfers large amounts of water resources from areas of production to far consumption regions, a phenomenon that has been named “the globalization of water” [11].

Virtual water trade is often considered as a solution to conditions of, overpopulation in regions with limited water availability, societal water stress, malnourishment and water wars [8]. Part of the global food security depends on virtual water trade [12], however, it has been noticed that virtual water trade has also negative implications, because, indirectly, it limits environmental stewardship [13], enhances inequality in the use of water resources [14], [15], and erodes societal resilience to drought [6]. Moreover, the trade of virtually embedded water is complicated by competitive, absolute and comparative advantage in the production and trade of food commodities [16], [17].

In the past two-to-three decades there has been an intensification of virtual water trade: the global volume of virtual water transfer and the number of links in the virtual water network (i.e., number of trade partnerships among countries) have almost doubled between 1986 and 2010, with an increasing dependence of the global virtual water trade on just a few exporting countries [18]. It is still unclear how the structural and functional properties of the virtual water network vary with the type of food commodity traded. Moreover, previous analyses [18], [19] looked at general trends in the volumes of virtual water traded and in the number of connections in, and topological characteristics of, the network [18]–[20] without explicitly evaluating how different countries and regions of the world are contributing to important structural changes in the network [21]. Understanding the factors and countries contributing to changes in the redistribution of virtual water resources is crucial to the assessment of how the system might respond to conditions of stress resulting for example from droughts. This paper investigates the geography of virtual water flows though an explicit analysis of the global spatiotemporal patterns of virtual water trade associated with plant, animal, luxury and other products. We investigate how some changes in the network of virtual water trade are correlated with the gross domestic product (GDP) and with combined metrics of life expectancy, education and income such as the Inequality adjusted Human Development Index (HDI).

## Methods

Detailed international trade data (FAOSTAT) for the period 1986–2010 from the Food and Agricultural Organization of the United Nations [22] were used to reconstruct the global trade patterns of food products reported by the FAOSTAT data base, including crops, crop-derived food commodities, and animal products. For each product, *m*, and year *t*, a trade matrix, ***T*** *_m_*(*t*) is generated, where the (*i,j*) element of the matrix is the export of that crop from country *i,* to country *j*. The reported trade data was rectified to political changes over the 25 year period to allow for clear trade node transitions (Appendix S1).

After the political rectification, by using for each food product the country-specific average blue and green virtual water content, *WC_m_*, [23], each individual crop trade matrix, ***T*** *_m_*(*t*), was converted to a crop specific virtual water trade matrix, ***C*** *_m_*(*t*) = *WC_m_*○***T*** *_m_*(*t*). The total virtual water trade matrix is simply ***C***(*t*) = Σ_m_ ***C*** *_m_*(*t*). ***C***(*t*) is non-symmetrical with two possible directed links between node *i*, and node *j*. The antisymmetrical undirected network matrix ***C*** *_sym_* (*t*) = ***C***(*t*)−***C***(*t*)***^T^*** thus defines the virtual water balance for each connection (*i,j*),with the balance for each year *t* and node *i*, defined as Σ_j_ ***C***(*i*, *j*, *t*). Virtual water contents for countries not reported in the estimates by [23] are based on the virtual water contents of the nearest neighbour (±10° of latitude and longitude). The global average values for the green and blue virtual water contents of that crop type were used for countries with no close neighbor.

Country-specific virtual water content estimates existed for 309 crops and animal products traded. Live animals were converted to live weight equivalents based on data from FAOSTAT [24]. The virtual water trade data were then further segregated into four primary groupings: edible crops, edible animal products, non-edible commodities (such as plant fibers, oils, oil cakes or animal hides) and luxury items (such as sugars, coffee and chocolate); the list of these commodities is detailed in Table S1. Populations of the nodes in the virtual water network over the period of study were obtained from Gapminder.org [25].

## Results

**Figure 1:**
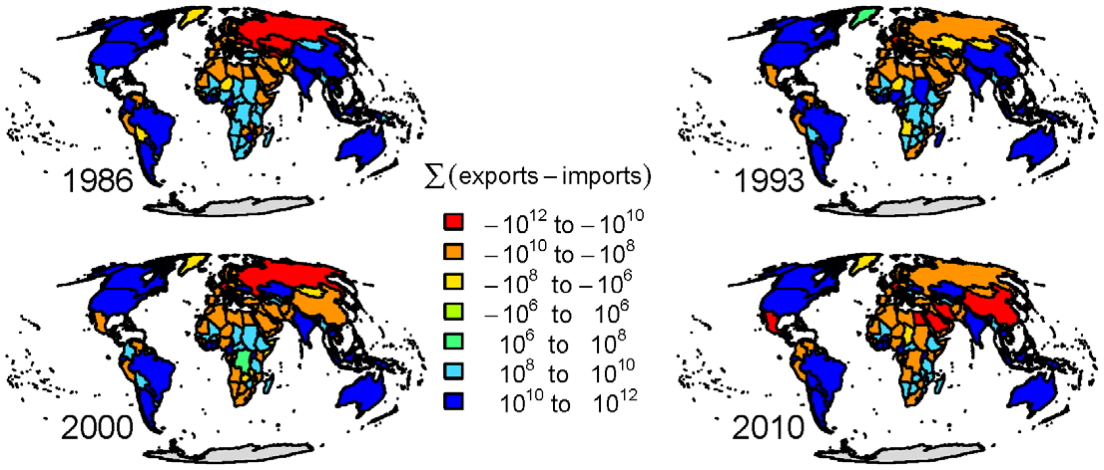
The virtual water import-export balance (m^3^) for the years 1986, 1993, 2000 and 2010. The total flux along the network has more than doubled, and the number of nodes net importing has risen from 127 to 149 with a corresponding decrease in net exporters from 104 to 72. As such the ratio of net importers to exporters has changed from 1.2 to 2.0.

There has been a strong increase in both the number of connections and total flux of virtual water over the 25 year period [18]. The discrepancy between net exporting countries and net importing countries has also increased with the ratio between the number of net importers per net exporter shifting from 1.32 in 1986 to 2. 5 in 2010 (Figure 1). Interestingly, in general countries either net export or net import a substantial amount of virtual water, while only few countries have smaller virtual water fluxes (e.g., in the range −10^6^ and 10^6^ m^3^). This gap persists even as countries shift in their virtual water import export balance from net importing to exporting or vice versa.

**Figure 2:**
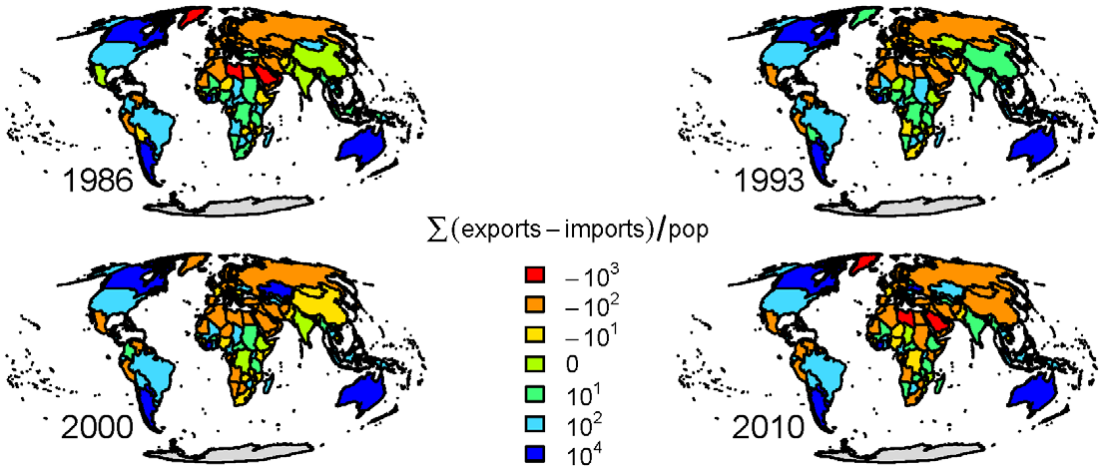
The virtual water import-export balance m^3^ per capita for the years 1986, 1993, 2000 and 2010.

Recasting the import-export balance by population we see a slightly different picture (Figure 2). In 1986 many countries are close to 0 in terms of export-import balance per capita. This shifts by 2010 with the majority of the global population net importing virtual water. In 1986 there were 0.57 net importing people per net exporter. By 2010 this switches to 1.43 people net importing per net exporter.

**Figure 3:**
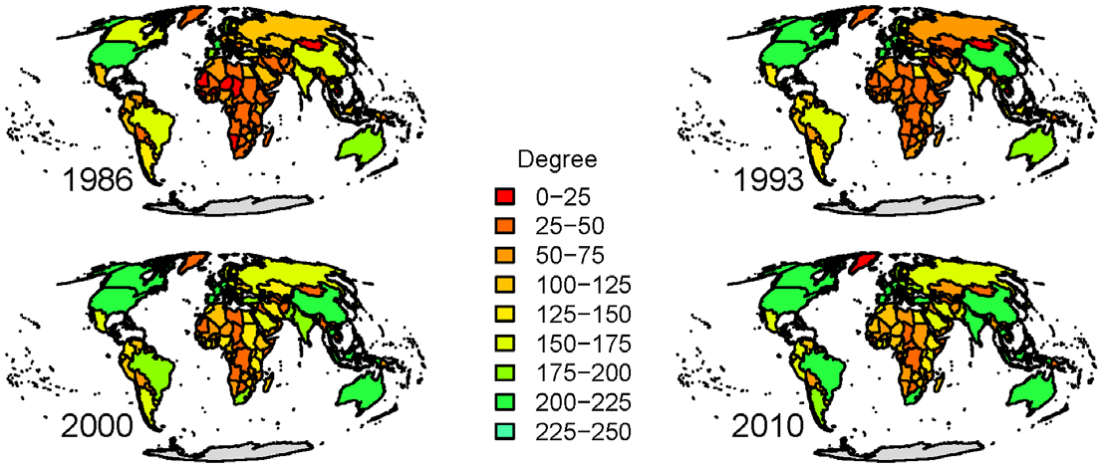
The degree (the sum of import and export connections) associated with each country for the years 1986, 1993, 2000, and 2010. While countries in general are more connected, this increase has not been as significant in most African countries.

Concurrent to the increase in total virtual water flux, the total number of connections that any country has (or degree), has increased over the 25 year period (Figure 3). This increase in connectivity and ‘globalization’ is less pronounced in the African continent, suggesting only limited participation to the global virtual water market, and more pronounced in the south Asian countries (China, India, Indonesia). The list of the top 5 net importing and exporting countries for the years 1986, 1993, 2000 and 2010 (Table 1) demonstrates the emergence of Brazil, Argentina, India, Indonesia and China as major players in the virtual water network. While most countries in the top 5 demonstrate an increase in strength (either net import or export), some countries within the top 5 such as Japan have demonstrated little (<5%) change in their net virtual water uptake over the 25 year period.

**Table 1:** The top 5 net importers (top) and exporters (bottom) and their virtual water export import balance (10^11^ m^3^) for the years 1986, 1993, 2000 and 2010.

Rank	1986 Balance		1993 Balance		2000 Balance		2010 Balance	
1	Japan	−0.77	Japan	−0.85	Japan	−0.88	China, mainland	−2.43
2	USSR	−0.54	Germany	−0.48	Germany	−0.49	Japan	−0.91
3	Italy	−0.37	Italy	−0.36	Italy	−0.45	Germany	−0.64
4	Germany	−0.32	Republic of Korea	−0.34	Russian Federation	−0.44	Iran	−0.58
5	United Kingdom	−0.23	Russian Federation	−0.29	Republic of Korea	−0.38	Italy	−0.55
1	United States of America	0.85	United States of America	1.04	United States of America	1.15	Brazil	1.87
2	Australia	0.60	Argentina	0.53	Argentina	0.99	United States of America	1.1.55
3	Argentina	0.44	Australia	0.40	Australia	0.79	Argentina	1.48
4	Thailand	0.34	Brazil	0.39	Brazil	0.58	Indonesia	0.84
5	Brazil	0.33	India	0.33	Canada	0.44	Canada	0.72

**Figure 4:**
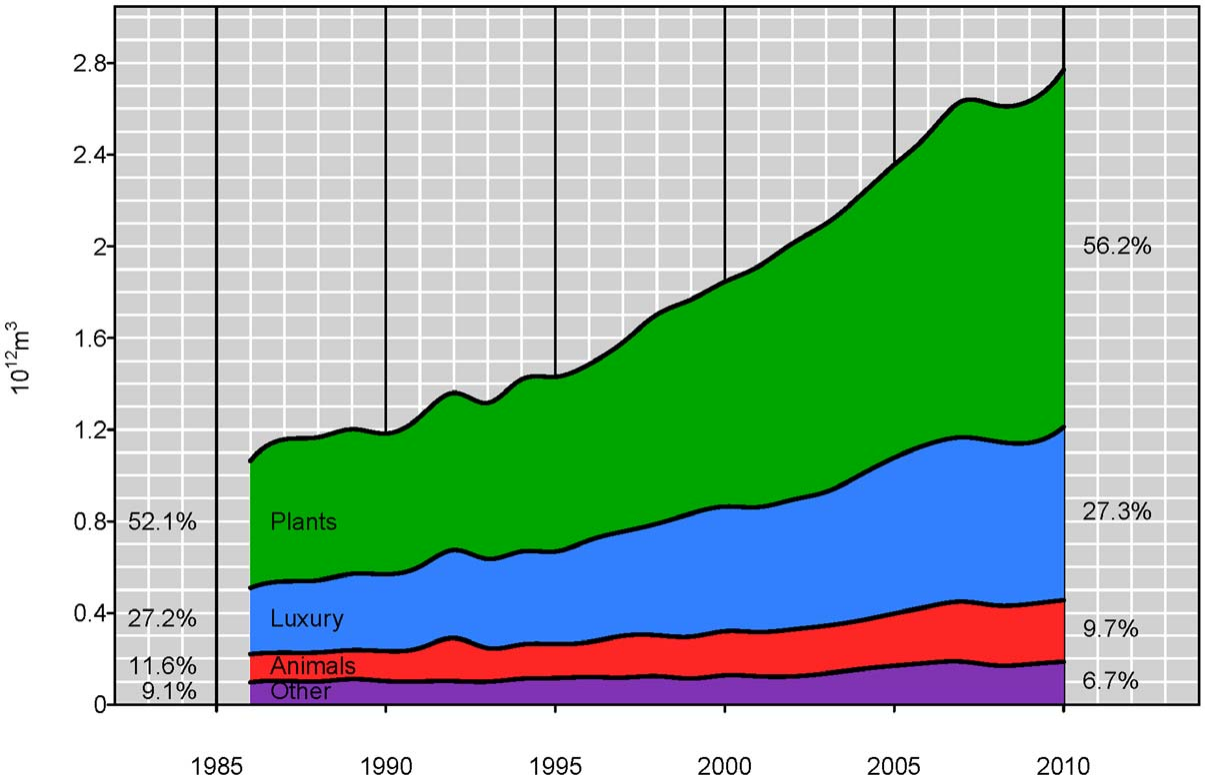
The increase in virtual water trade and the percentage of the total virtual water flux in the network corresponding to plant, animals, luxury, and other commodities. While there is an increase in the virtual water flux associated with all commodity categories, the fraction of the total flux associated with animals and other has decreased. There is a corresponding almost tripling of crop products and luxury products over the 25 year period with animal products and fibers increasing to a lesser extent.

Separation of products into edible plant based products, edible animal based products, luxury products and other plant and animal derived products allows for examining the consumption change in virtual water as related to changes in commodity trade. The top 15 commodities by virtual water volume for the years 1986, 1993, 2000, and 2010, demonstrate the maintenance of cereal grains, oils and cotton lint being the predominant traded products (Table 2). However, luxury items such as coffee and chocolate remain consistently in the top 15 throughout the period and there is an increase in the virtual water trade associated with bovine meat. The virtual water volume associated with the top 15 products accounts for a relatively constant percentage (≈66%) of the total volume from 1986 to 2010. The remaining third of the flux is spread among 294 different commodities. While trade of virtual water associated with all commodities has increased (Figure 4), the increase in the virtual water trade associated with edible plant based products and luxury commodities is not matched by a comparable increases in the virtual water trade in edible animal products and other plant and animal derived products. The flux of virtual water associated with edible plant and luxury items has more than doubled over the 25 year period. The virtual water flux associated with edible animal products and other commodities has “only” doubled (Figure 3). Interestingly, the proportion of virtual water associated with the four commodity type groupings has remained relatively constant over the years with small increase associated with edible plant and luxury items and a corresponding decrease in animal and non-edible products.

**Table 2:** The top 20 products and corresponding virtual water flux (10^11^ m^3^) for the years 1986, 1993, 2000 and 2010.

Rank	1986		1993		2000		2010	
**1**	Wheat	1.34	Wheat	1.45	Wheat	1.93	Wheat	2.30
**2**	Coffee, green	0.80	Coffee, green	0.89	Chocolate Prsnes	1.25	Soybeans	2.29
**3**	Cake of Soybeans	0.59	Cake of Soybeans	0.87	Soybeans	1.22	Chocolate Prsnes	2.02
**4**	Soybeans	0.56	Chocolate Prsnes	0.81	Cake of Soybeans	1.08	Cake of Soybeans	1.81
**5**	Maize	0.49	Soybeans	0.70	Coffee, green	0.99	Palm oil	1.56
**6**	Cotton lint	0.45	Cocoa beans	0.57	Cocoa beans	0.72	Maize	1.11
**7**	Cocoa beans	0.42	Maize	0.51	Maize	0.66	Coffee, green	1.04
**8**	Sugar Raw Centrifugal	0.36	Cotton lint	0.46	Palm oil	0.61	Cocoa beans	0.91
**9**	Chocolate Prsnes	0.34	Palm oil	0.37	Cotton lint	0.56	Cotton lint	0.83
**10**	Palm oil	0.26	Meat-Cattle Boneless(Beef & Veal)	0.35	Soybean oil	0.53	Meat-Cattle Boneless(Beef & Veal)	0.75
**11**	Sugar Refined	0.25	Sugar Refined	0.32	Meat-Cattle Boneless(Beef & Veal)	0.52	Rice Milled	0.69
**12**	Rice Milled	0.23	Rice Milled	0.32	Sugar Refined	0.50	Soybean Oil	0.68
**13**	Meat-Cattle Boneless(Beef & Veal)	0.23	Soybean oil	0.30	Rice Milled	0.45	Sugar Refined	0.6
**14**	Barley	0.21	Cocoa Butter	0.29	Cocoa Butter	0.41	Cocoa Butter	0.54
**15**	Soybean oil	0.20	Sugar Raw Centrifugal	0.22	Sugar Raw Centrifugal	0.38	Sugar Raw Centrifugal	0.52

**Figure 5:**
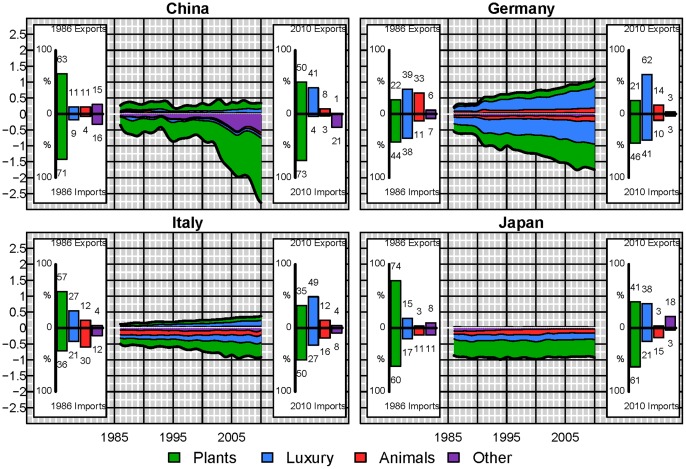
Detailed virtual water trade (10^11^ m^3^) by commodity type for China (mainland), Germany, Italy and Japan. Positive values refer to exports and negative values to imports. The insets indicate the percentage of virtual water imports or exports contributed by plant, animal, luxury and other products.

**Figure 6:**
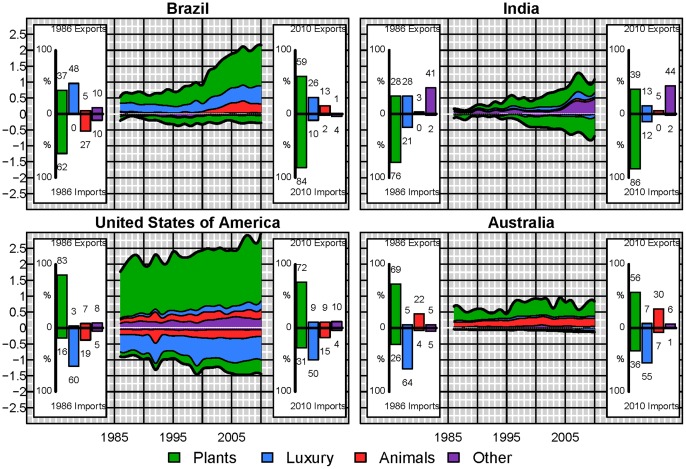
Detailed virtual water trade (10^11^ m^3^) by commodity type for Brazil, India, The United States of America and Australia. Positive values refer to exports and negative values to imports. The insets indicate the percentage of virtual water imports or exports contributed by plant, animal, luxury and other products.

Even though, globally, these proportions remain almost constant, at the country scale there have been important changes. Looking at a selection of some of the major player in the virtual water network (Table 1) we see distinct behavior differences (Figure 5 and 6). For example, in 1986 China was a net virtual water exporter with exports including other non-edible commodities and 9% of their virtual water imports associated with luxury items. By 2010, virtual water imports associated with non-edible plant and animal commodities comprise 214% of China’s imports, and luxury items comprise 41% of the virtual water exports with the top 2 imported commodities in terms of virtual water being soybeans and cotton lint. China currently dominates not just the global virtual water imports, but specifically the virtual water associated with non-edible plant and animal commodities (31% of all global virtual water trade associated with non-edible plant and animal commodities). Turkey was the next largest importer of non-edible plant and animal products accounting for only 5% followed by Italy with ∼3%. Similarly, China was importing 13% of all virtual water associated with edible plant products. This is contrasted by a country such as Japan (Figure 6), whose virtual water imports by commodity type have remained almost constant over the two and a half decades. These two countries are in turn contrasted by Germany and Italy. Like Japan, the proportional make up of Germany’s virtual water imports has remained almost constant over the 25 year period; however, it has significantly decreased its meat exports while increasing its luxury product exports. Since 1991 and reunification, Germany has more than doubled its importation of virtual water, while not quite doubling its virtual water exports. Similarly Italy has more than doubled its importation of virtual water, however, it has shifted toward importing more virtual water associated with edible plant commodities and increased exportation of luxury items.

Examining a few of the major net exporters of virtual water (Figure 6), the United States of America continue to be a major importer of luxury products and the primary importer of virtual water associated with animal based products. By 2010 the USA accounts for ∼8.5% of the virtual water trade in animal products, while the Russian federation accounts for 8%. Germany, Japan and Italy account each for ∼ 5% of global virtual water imports in animal product. The United States of America and Germany are the largest importers of virtual water associated with luxury items each comprising 9% of the global luxury commodity virtual water imports. However, whereas virtual water exports from Germany do not exceed their heavy importation of virtual water, the import of virtual water associated with meat and luxury products in the United States of America is easily compensated for by the large export of plant based commodities, predominantly soybeans, wheat and maize. While the United States of America is not the largest net exporter of virtual water in 2010, they are the largest exporter of virtual water with a total exported volume of ∼10^11^ m^3^. The large increases in the trade from the United States of America has not matched the changes that have occurred in the trade from Brazil and India. The virtual water exports from Brazil are three times their virtual water exports in 1986, whereas virtual water exports from India in 2010 are almost 5 times greater than those in 1986 (Figure 6). Australia in contrast has shown large variability in its exportation of virtual water associated with plant based commodities matching well with Australian precipitation records (Figure 6). These large discrepancies between commodity based imports and exports of virtual water for individual nodes indicate that, while globally the ratio of virtual water associated with the four commodity groups has changed only slightly over the period of study, virtual water trade in individual nodes is highly variable.

**Figure 7:**
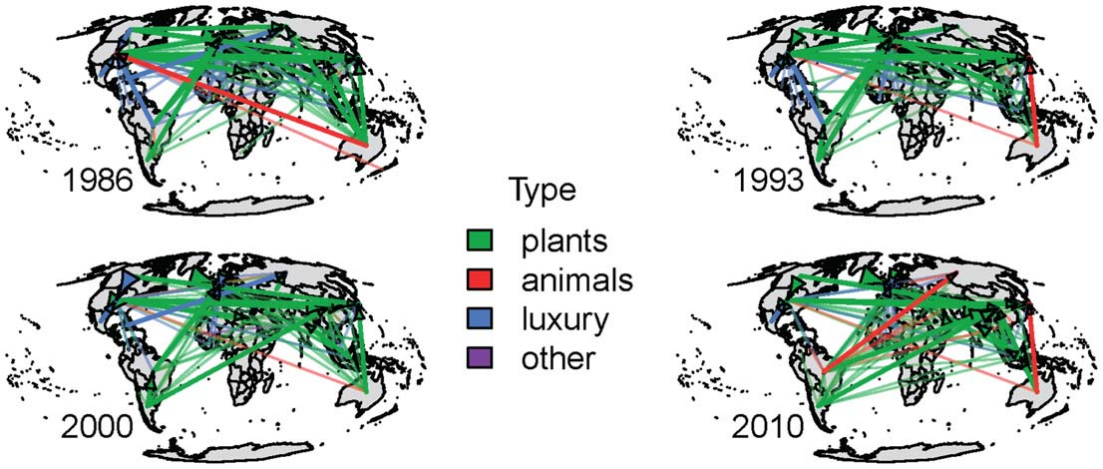
The Network associated with the top 50% of the virtual water flux for the years 1986, 1993, 2000 and 2010. Color in the links based on the most prevalent commodity along the link in terms of total flux through the link. The heavy lines indicate the links which comprise the top 25% of the virtual water flux.

Figure 7 shows the top 50% of the virtual water fluxes (top 25% in heavy lines). It reveals a dynamic network, both in terms of connections as well as dominant flux along connections. Here each link is colored based on the commodity associated with the largest virtual water volume moving through that link. While there is a change, both in the links and major commodity along links, there is some consistency in the importation of virtual water associated with meat products into the United States and exportation of plant products from North and South America to Asia and Europe. Examining the net importers and exporters amongst the four commodity networks revels that major players in the global virtual water network are not necessarily a major contributor in all the commodity networks (Table 3). Moreover, the fluxes of virtual water moving into or out of Africa are consistently relatively small. However, the per capita export-import balance (Figure 2) of African nations is not as disparate in comparison to the rest of the world. In contrast, Australia and Argentina stand out as large per capita exporters with the Arabian Peninsula, and some of Europe being the largest net importers. Even though China is the largest net importer, its per capita imports are moderate.

**Table 3:** The top 5 net importers (left) and net exporters (right) and their virtual water export import balance (10^11^ m^3^) for the year 2010 in the four commodity type networks.

	Net importing	Net imports	Net Exporting	Net exports
**Plant**	China, mainland	−1.85	United States of America	1.71
	Germany	−0.56	Argentina	1.42
	Japan	−0.56	Brazil	1.04
	Egypt	−0.37	Canada	0.65
	Italy	−0.32	Indonesia	0.61
**Animal**	Russian Federation	−0.21	Brazil	0.27
	Japan	−0.14	Australia	0.24
	Italy	−0.10	New Zealand	0.11
	China, Hong Kong SAR	−0.07	Canada	0.09
	United Kingdom	−0.53	Denmark	0.08
**Luxury**	United States of America	−0.47	Brazil	0.54
	United Kingdom	−0.21	Indonesia	0.32
	Russian Federation	−0.20	Netherlands	0.27
	Japan	−0.19	Cote d’Ivoire	0.21
	Spain	−0.12	Ghana	0.18
**Other**	China, mainland	−0.58	India	0.37
	Turkey	−0.09	United States of America	0.20
	Italy	−0.06	Australia	0.04
	Republic of Korea	−0.05	Canada	0.04
	Indonesia	−0.05	Uzbekistan	0.03

Thus, while there has been a large increase in the virtual water flux over the 25 year period, the water burden (i.e., demand for virtual water exports) has been placed on a smaller and smaller portion of the world population. In 1986 68% of the world population was in countries that were net exporters of water. In the late 1980’s China and India both were briefly net importers and the fraction of the world population net importing sharply increased. However, India has slowly become a larger net exporter of virtual water while China has continued to increase its virtual water imports. As a result, by the mid 1990’s over half the world’s population lived in countries that net imported virtual water. By 2010 roughly 60% of the world population lived in net importing countries. By weighting the population of each country *i* by what fraction of their virtual water exports is transferred to country *j*, the proportion of the population of country *i* that is supporting country *j*, 
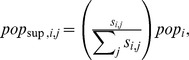
 can be calculated where *s_i,j_* is the strength of the link from country *i* to country *j*. Repeating for all countries, and scaling by the global population allows for examination of the fraction of the global population that is supplying virtual water to each given country. In general, any particular country obtains its imports from less than 9% of the global population. By only considering those countries that net import virtual water, we find that any of these individual countries are supplied by less than 4% of the global population. Sorting the net importers by their export-import balance and looking at the populations supplying those countries shows that 32% of the global population is providing 90% of the virtual water, while 13% is providing 50% of the virtual water imported by net importers. All commodities show a similar pattern, except for the virtual water traded with animal and luxury products, which is supplied by a smaller fraction of the global population.

To investigate the drivers of virtual water trade, the import strength, 
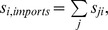
 was related to the Inequality adjusted Human Development Index (IHDI) [26] through a power law relationship (s_i,imports_ = a IHDI_i_^b^ ). IHDI data is limited to 160 nodes in the network, with no preferential influence of nodal population. This relationship was found to be stronger for the luxury and animal product network (Table 4).

**Table 4:** Parameters obtained by fitting a power law to import strength and IHDI values (s_i,imports_ = a IHDI_i_^b^).

IMPORT	b	a(m^3^)	r^2^
Full	10.31	2.20	0.29
Plant	9.91	1.76	0.16
Animal	9.32	3.07	0.39
Luxury	9.93	3.25	0.44
Other	8.90	3.46	0.26

## Discussion and Conclusions

The strong increase in virtual water trade over the past 25 year period is not as ubiquitous as the term “globalization” implies. Rather, there are distinct increases of virtual water flows associated with a few countries, while the majority of countries experienced only smaller fluctuations.

Overall, the results presented in this study indicate in multiple ways that there is a small global population controlling large amounts of the virtual water exports [18]. This indicates potential for limited resilience of the virtual water network with respect to the possible decision of one of these suppliers to stop or reduce its exports. Most of the virtual water resources available for trade tend to remain concentrated in a small set of countries, controlled by a small fraction of the global populations similar to the Pareto principle. Thus, the importing countries are left with limited options for the diversification of their virtual water providers.

The controls on the strengths in both the total virtual water network, and the commodity networks remain poorly understood. There is a dependency of import strengths on the Inequality adjusted Human Development Index. This relationship is stronger in the case of luxury and animal products (Table 3), consistent with the so-called Bennett’s Law, which postulates that diets become richer in fats, protein, and sugar as societies become wealthier [27]. In other words, the import of luxury and animal products is more intense in those countries who can afford them, which explains the stronger dependence of virtual water traded in animal and luxury products on the social development status. This indicates that the drivers of the full virtual water network are not only multi-factorial, but that they vary depending on the commodity classes. Other indices (e.g., Grass Domestic Product (GDP)) exhibited a weaker relation with import strength. Since IHDI incorporates many factors including gross national income, mortality rates, and education, it may allow for better cross comparison of countries. However, IHDI does not include any metric of water resource availability for the individual countries. These results are echoed in the findings of the community structure results of D’Odorico et al. [28], which demonstrated that the community structure of the virtual water network appears to not align well with any one factor (e.g., population, GDP, distance metrics or production).

**Figure 8:**
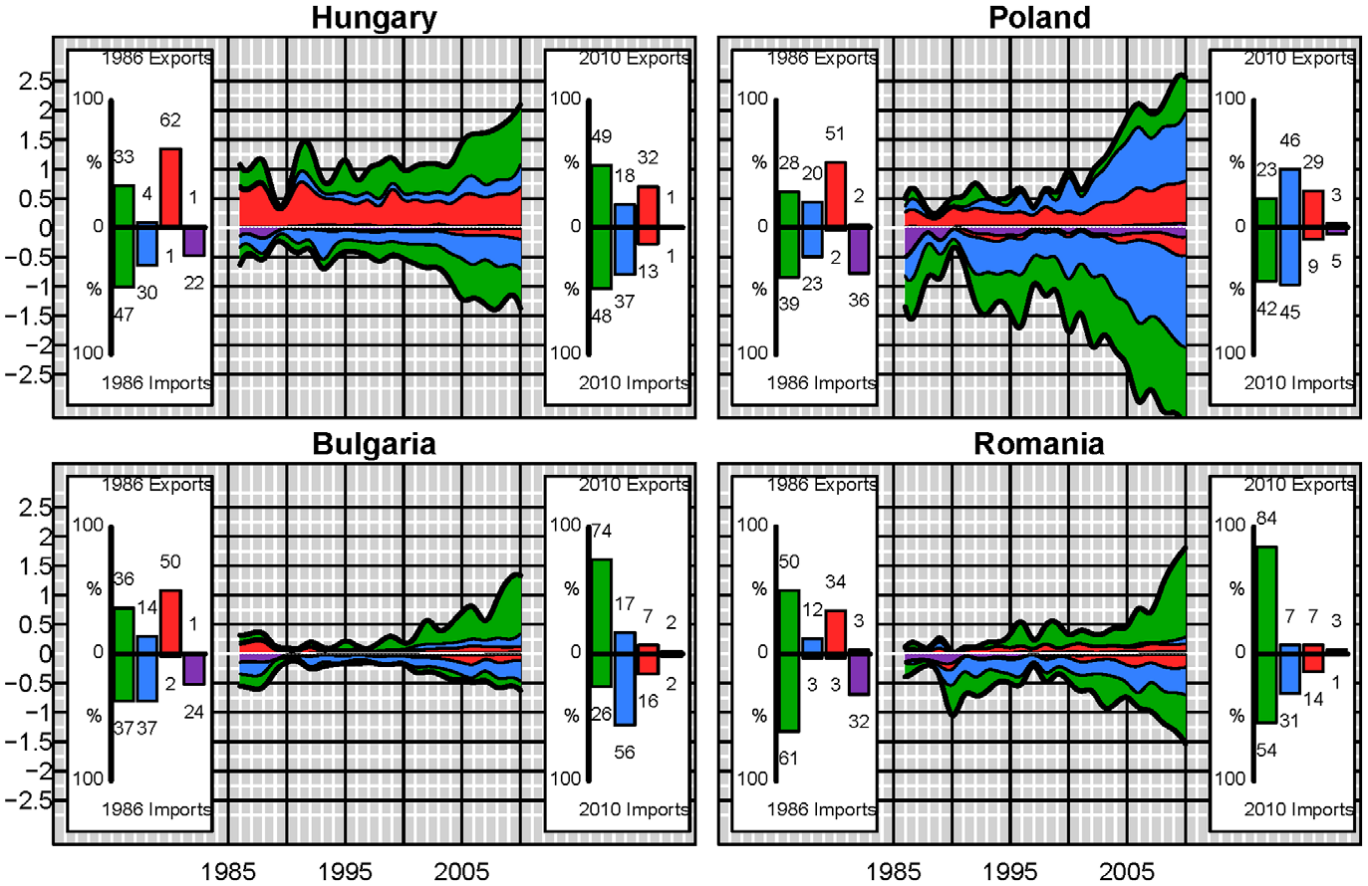
Detailed virtual water trade (10^10^ m^3^) by commodity type for Hungary, Poland, Bulgaria and combined former USSR and Russian federation. The insets indicate the percentage of virtual water imports or exports contributed by plant, animal, luxury and other products.

Prior analysis has shown regional contributions to virtual water fluxes and their changes over time [19], however strong changes of individual countries rather than regions seem to be controlling the patterns and changes in the virtual water network. For example in 1992 there is globally a spike in the virtual water associated with the trade of animal products (Figure 4). This spike is also apparent in the temporal virtual water trade of the United States of America (Figure 6) and is due to a large increase in imports of cattle and pig meat from Mexico. Some of this results from the post cold war subsidy reforms which led to a significant decrease in livestock production [29] in the Russian Federation and other eastern European countries. This reform was combined with flooding in 1991 in China resulting in decreases in pig production [29]. Along with this potential increase in production, beef exports from the United States of America rapidly increased beginning in 1992. The corresponding spike in meat imports in the United States in 1992 was due to a net importation of pig meat in 1992. Alternately in 2003, the first reported case of mad cow disease in the United States of America resulted in significant decrease in meat exports (Figure 6). This change is not apparent in the global network (Figure 4). A sudden decline in importation and exportation of VW in the Eastern Bloc countries (Hungary, Poland, Romania and Bulgaria) (Figure 8) occurred around the 1990 fall of the Iron curtain, and resulted in a small dip in the global trade of VW (Figure 4), which was then followed by a substantial increase in VW importation, particularly in luxury items. Thus, while some of these node-specific changes relate to regional dynamics causing regional-scale changes, others are more global in scale. As such the resilience of the virtual water network to socioeconomic, political and environmental changes still needs to be explored.

While the proportional constituency in terms of commodity type of virtual transfers has remained relatively unchanged over two decades, the changes in virtual water proportions in any given node is variable. Moreover, the major contributors to the change in the virtual network do not seem to be responsible for the consistency in these global proportions and virtual water exports and imports do not seem to be driven by any single factor, but rather depend on multitude political, socio-economic and geographic factors.

## Supporting Information

Appendix S1 **Political data rectification.**
(DOCX)

Table S1 **Commodities used in this study and categorization.**
(DOCX)

## References

[1] Gleick PH (1999) The human right to water: Pacific Institute for Studies in Development, Environment, and Security.

[2] Mason MB, Lyn KG, Quezon AU, Jamie B (2012) Implementing an evolving human right through water and sanitation policy. 10.2166/wp.2012.198 PMC7006955 32038095

[3] Gleick PH (1993) Water and conflict: Fresh water resources and international security. International security: 79–112.

[4] Wolf AT (1999) “Water Wars” and Water Reality: Conflict and Cooperation Along International Waterways. NATO SCIENCE SERIES 2 ENVIRONMENTAL SECURITY 65: 251–268.

[5] Morison JIL, Baker NR, Mullineaux PM, Davies WJ (2008) Improving water use in crop production. Philosophical Transactions of the Royal Society B: Biological Sciences 363: 639–658. 10.1098/rstb.2007.2175 PMC2610175 17652070

[6] D’Odorico P, Laio F, Ridolfi L (2010) Does globalization of water reduce societal resilience to drought? Geophysical Research Letters 37.

[7] Schade C, Pimentel D (2010) Population crash: prospects for famine in the twenty-first century. Environment, Development and Sustainability 12: 245–262.

[8] Allan JA (1998) Virtual water: A strategic resource global solutions to regional deficits. Ground Water 36: 545–546.

[9] Allan T, editor (1993) Fortunately there are substitutes for water: otherwise our hydropolitical futures would be impossible, in Proceedings of the Conference on Priorities for Water Resources Allocation and Management,. Southampton, U. K: Overseas Dev. Agency. 13–26 p.

[10] Hoekstra A (2002) Virtual water trade: Proceedings of the international expert meeting on virtual water trade. Delft, Netherlands: UNESCO‐IHE.

[11] Hoekstra A, Chapagain AK (2008) Globalization of water: Sharing the planet’s freshwater resources. Oxford, UK: Blackwell Publishing.

[12] Mekonnen MM, Hoekstra AY (2011) The green, blue and grey water footprint of crops and derived crop products. Hydrology and Earth System Sciences 15: 1577–1600.

[13] Carr J, D’Odorico P, Laio F, Ridolfi L, Seekell D (2012) Inequalities in the networks of virtual water flow. *Eos Trans AGU* 93.

[14] Seekell DA (2011) Does the Global Trade of Virtual Water Reduce Inequality in Freshwater Resource Allocation? Society & Natural Resources 24: 1205–1215.

[15] Seekell DA, D’Odorico P, Pace ML (2011) Virtual water transfers unlikely to redress inequality in global water use. Environmental Research Letters 6.

[16] Wichelns D (2004) The policy relevance of virtual water can be enhanced by considering comparative advantages. Agricultural Water Management 66: 49–63.

[17] Dan W (2009) Optimization of water conservancy industry structure based on comparative advantage of water resources [J]. Journal of Economics of Water Resources 3: 019.

[18] Carr JA, D’Odorico P, Laio F, Ridolfi L (2012) On the temporal variability of the virtual water network. Geophysical Research Letters 39.

[19] Dalin C, Konar M, Hanasaki N, Rinaldo A, Rodriguez-Iturbe I (2012) Evolution of the global virtual water trade network. Proceedings of the National Academy of Sciences of the United States of America 109: 5989–5994. 22474363 10.1073/pnas.1203176109 PMC3341016

[20] Konar M, Dalin C, Suweis S, Hanasaki N, Rinaldo A (2011) Water for food: The global virtual water trade network. Water Resources Research 47. 10.1073/pnas.1203176109 PMC3341016 22474363

[21] Suweis S, Konar M, Dalin C, Hanasaki N, Rinaldo A (2011) Structure and controls of the global virtual water trade network. Geophysical Research Letters 38.

[22] Food and Agriculture Organization of the United Nations Website. Available: http://faostat3.fao.org/home/index.html Accessed 2012 Oct 1.

[23] Mekonnen MM, Hoekstra AY (2010) A global and high-resolution assessment of the green, blue and grey water footprint of wheat. Hydrology and Earth System Sciences 14: 1259–1276.

[24] Food and Agriculture Organization of the United Nations Website. Available: http://www.fao.org/fileadmin/templates/ess/documents/methodology/tcf.pdf Accessed 2012 Feb 10.

[25] Gapminder website. Available: http://www.gapminder.org. Accessed 2012 Aug 5.

[26] Human Development Report Website. Available http://hdr.undp.org/en/statistics/ihdi/Accessed 2012 May 18.

[27] (2011) Foresight. The Future of Food and Farming. London: The Government Office for Science.

[28] D’Odorico P, Carr J, Laio F, Ridolfi L (2012) Spatial organization and drivers of the virtual water trade: A community-structure analysis. *Environmental Research Letters* 7.

[29] United States department of Agriculture Website Available: http://www.ers.usda.gov/publications/aer813/aer813c.pdf Accessed 2012 May 20.

