# Supplementary material for: Recent History and Geography of Virtual Water Trade
Source: PLoS One. 2013 Feb 15;8(2):e55825. doi: 10.1371/journal.pone.0055825 (PMC3574060; doi:10.1371/journal.pone.0055825)
Supplement: Appendix S1 — Political data rectification. (DOCX) [file pone.0055825.s001.docx]

**Appendix S1.** Political data rectification:

Between 1987 and 1991, due to regime change, Ethiopia was renamed the Peoples Democratic Republic of Ethiopia. This did not create a new distinct trading entity and as such between 1986 and 1991, all trade reported to Ethiopia and the Peoples Democratic Republic of Ethiopia was merged. In 1993 Eritrea emerged as a distinct political node, as such, prior to 1993, all trade reported to Eritrea was merged into trade to Ethiopia. After 1990, any trade reported to The Federated Republic of Germany or German Democratic Republic was combined into Germany. From 1990 onward all trade prescribed to North and South Yemen was combined into Yemen corresponding to the political merging. Prior to 1991 all trade associated with Yugoslavia was combined into a single country, Yugoslav SFR. Slovenia, The former Yugoslav Republic of Macedonia and Croatia declared independence in the Fall of 1991. As such, reported trade to Yugoslav SFR from 1992 onward was merged into Serbia and Montenegro. In 1992, Bosnia and Herzegovina, Slovenia, Serbia and Montenegro, The former Yugoslav Republic of Macedonia and Croatia were considered politically distinct, and thus were considered as possible nodes in the network even if no trade was reported for that year. Between 1992 and 2006 any distinct reporting to Serbia and/or Montenegro was merged into Serbia and Montenegro. From 2006 and onwards “Serbia” and “Montenegro” were treated independently. Prior to 1991, all former reported trade to and from the former soviet countries, was attributed to USSR. After 1991 those former soviet states become possible active nodes in the network. Any trade reported to USSR after 1991 was prescribed to the Russian Federation. Prior to 1993 all trade to the Czech Republic and Slovakia was attributed to Czechoslovakia. After 1993, and trade to Czechoslovakia, was prescribed to Czech Republic. Prior to 2000, trade reported Belgium-Luxembourg was split between Belgium and Luxembourg using the mean virtual water ratio between Belgium and Luxembourg for 2001 through 2010.
