# Supplementary material for: Recent History and Geography of Virtual Water Trade
Source: PLoS One. 2013 Feb 15;8(2):e55825. doi: 10.1371/journal.pone.0055825 (PMC3574060; doi:10.1371/journal.pone.0055825)
Supplement: Table S1 — Commodities used in this study and categorization. (DOCX) [file pone.0055825.s002.docx]

**Table S2.**  Commodities used in this study and categorization

| **id** | **Commodity** | **Category** |
| --- | --- | --- |
| 15 | Wheat | plant |
| 16 | Flour of Wheat | plant |
| 18 | Macaroni | plant |
| 20 | Bread | plant |
| 27 | Rice, paddy | plant |
| 28 | Rice Husked | plant |
| 31 | Rice Milled | plant |
| 32 | Rice Broken | plant |
| 38 | Rice Flour | plant |
| 44 | Barley | plant |
| 46 | Barley Pearled | plant |
| 48 | Barley Flour and Grits | plant |
| 49 | Malt | plant |
| 56 | Maize | plant |
| 58 | Flour of Maize | plant |
| 60 | Maize oil | plant |
| 71 | Rye | plant |
| 72 | Flour of Rye | plant |
| 75 | Oats | plant |
| 76 | Oats Rolled | plant |
| 79 | Millet | plant |
| 83 | Sorghum | plant |
| 89 | Buckwheat | plant |
| 92 | Quinoa | plant |
| 94 | Fonio | plant |
| 97 | Triticale | plant |
| 101 | Canary seed | plant |
| 103 | Mixed grain | plant |
| 108 | Cereals, nes | plant |
| 116 | Potatoes | plant |
| 117 | Potatoes Flour | plant |
| 118 | Frozen Potatoes | plant |
| 120 | Potato Offals | plant |
| 121 | Tapioca of Potatoes | plant |
| 122 | Sweet potatoes | plant |
| 125 | Cassava | plant |
| 126 | Flour of Cassava | plant |
| 127 | Tapioca of Cassava | plant |
| 128 | Cassava Dried | plant |
| 129 | Cassava Starch | plant |
| 135 | Yautia (cocoyam) | plant |
| 136 | Taro (cocoyam) | plant |
| 137 | Yams | plant |
| 149 | Roots and Tubers, nes | plant |
| 150 | Flour of Roots and Tubers | plant |
| 176 | Beans, dry | plant |
| 181 | Broad beans, horse beans, dry | plant |
| 187 | Peas, dry | plant |
| 191 | Chick peas | plant |
| 195 | Cow peas, dry | plant |
| 197 | Pigeon peas | plant |
| 201 | Lentils | plant |
| 203 | Bambara beans | plant |
| 205 | Vetches | plant |
| 210 | Lupins | plant |
| 211 | Pulses, nes | plant |
| 212 | Flour of Pulses | plant |
| 216 | Brazil nuts, with shell | plant |
| 217 | Cashew nuts, with shell | plant |
| 220 | Chestnuts | plant |
| 221 | Almonds, with shell | plant |
| 222 | Walnuts, with shell | plant |
| 223 | Pistachios | plant |
| 224 | Kolanuts | plant |
| 225 | Hazelnuts, with shell | plant |
| 231 | Almonds Shelled | plant |
| 232 | Walnuts Shelled | plant |
| 233 | Hazelnuts Shelled | plant |
| 234 | Nuts, nes | plant |
| 235 | Prepared Nuts (Exc.Groundnuts) | plant |
| 236 | Soybeans | plant |
| 237 | Soybean oil | plant |
| 238 | Cake of Soybeans | plant |
| 239 | Soya Sauce | plant |
| 240 | Soya Paste | plant |
| 241 | Soya curd | plant |
| 242 | Groundnuts, with shell | plant |
| 243 | Groundnuts Shelled | plant |
| 244 | Groundnut oil | plant |
| 249 | Coconuts | plant |
| 251 | Copra | plant |
| 252 | Coconut (copra) oil | plant |
| 256 | Palm kernels | plant |
| 257 | Palm oil | plant |
| 258 | Palm kernel oil | plant |
| 259 | Cake of Palm Kernel | plant |
| 260 | Olives | plant |
| 261 | Olive oil, virgin | plant |
| 262 | Olives Preserved | plant |
| 263 | Karite Nuts (Sheanuts) | plant |
| 264 | Butter of Karite Nuts | plant |
| 267 | Sunflower seed | plant |
| 268 | Sunflower oil | plant |
| 270 | Rapeseed | plant |
| 271 | Rapeseed oil | plant |
| 280 | Safflower seed | plant |
| 289 | Sesame seed | plant |
| 290 | Sesame oil | plant |
| 292 | Mustard seed | plant |
| 296 | Poppy seed | plant |
| 299 | Melonseed | plant |
| 331 | Cottonseed oil | plant |
| 336 | Hempseed | plant |
| 339 | Oilseeds, Nes | plant |
| 340 | Oil of vegetable origin, nes | plant |
| 358 | Cabbages and other brassicas | plant |
| 366 | Artichokes | plant |
| 367 | Asparagus | plant |
| 372 | Lettuce and chicory | plant |
| 373 | Spinach | plant |
| 388 | Tomatoes | plant |
| 389 | Tomatojuice Concentrated | plant |
| 390 | Juice of Tomatoes | plant |
| 391 | Paste of Tomatoes | plant |
| 392 | Tomato Peeled | plant |
| 393 | Cauliflowers and broccoli | plant |
| 394 | Pumpkins, squash and gourds | plant |
| 397 | Cucumbers and gherkins | plant |
| 399 | Eggplants (aubergines) | plant |
| 401 | Chillies and peppers, green | plant |
| 402 | Onions (inc. shallots), green | plant |
| 403 | Onions, dry | plant |
| 406 | Garlic | plant |
| 414 | Beans, green | plant |
| 417 | Peas, green | plant |
| 423 | String beans | plant |
| 426 | Carrots and turnips | plant |
| 430 | Okra | plant |
| 446 | Maize, green | plant |
| 447 | Sweet Corn Frozen | plant |
| 460 | Veg.Prod.Fresh Or Dried | plant |
| 461 | Carobs | plant |
| 463 | Vegetables fresh nes | plant |
| 466 | Juice of Vegetables Nes | plant |
| 469 | Vegetables Dehydrated | plant |
| 471 | Vegetables in Vinegar | plant |
| 472 | Vegetables Preserved Nes | plant |
| 473 | Vegetable Frozen | plant |
| 486 | Bananas | plant |
| 489 | plantains | plant |
| 490 | Oranges | plant |
| 491 | Orange juice, single strength | plant |
| 495 | Tangerines, mandarins, clem. | plant |
| 497 | Lemons and limes | plant |
| 507 | Grapefruit (inc. pomelos) | plant |
| 509 | Juice of Grapefruit | plant |
| 512 | Citrus fruit, nes | plant |
| 513 | Citrus juice, single strength | plant |
| 515 | Apples | plant |
| 517 | Cider Etc | plant |
| 518 | Apple juice, single strength | plant |
| 521 | Pears | plant |
| 526 | Apricots | plant |
| 527 | Dry Apricots | plant |
| 530 | Sour cherries | plant |
| 531 | Cherries | plant |
| 534 | Peaches and nectarines | plant |
| 536 | Plums and sloes | plant |
| 537 | Plums Dried (Prunes) | plant |
| 544 | Strawberries | plant |
| 547 | Raspberries | plant |
| 549 | Gooseberries | plant |
| 550 | Currants | plant |
| 552 | Blueberries | plant |
| 554 | Cranberries | plant |
| 558 | Berries Nes | plant |
| 560 | Grapes | plant |
| 561 | Raisins | plant |
| 562 | Grape Juice | plant |
| 567 | Watermelons | plant |
| 568 | Other melons (inc.cantaloupes) | plant |
| 569 | Figs | plant |
| 571 | Mangoes, mangosteens, guavas | plant |
| 572 | Avocados | plant |
| 574 | Pineapples | plant |
| 576 | Juice of Pineapples | plant |
| 577 | Dates | plant |
| 591 | Cashew apple | plant |
| 592 | Kiwi fruit | plant |
| 600 | Papayas | plant |
| 603 | Fruit, tropical fresh nes | plant |
| 604 | Fruit Tropical Dried Nes | plant |
| 619 | Fruit Fresh Nes | plant |
| 622 | Fruit Juice Nes | plant |
| 748 | Peppermint | plant |
| 1232 | Food Prep Nes | plant |
| 866 | Cattle | animal |
| 867 | Cattle meat | animal |
| 868 | Offals of Cattle, Edible | animal |
| 870 | Meat-CattleBoneless(Beef&Veal) | animal |
| 872 | Meat of Beef,Drd, Sltd,Smkd | animal |
| 874 | Sausage Beef&Veal | animal |
| 882 | Cow milk, whole, fresh | animal |
| 886 | Butter Cow Milk | animal |
| 888 | Milk Skm of Cows | animal |
| 889 | Milk Whole Cond | animal |
| 890 | Whey Condensed | animal |
| 891 | Yoghurt | animal |
| 893 | Butterm.,Curdl,Acid.Milk | animal |
| 897 | Milk Whole Dried | animal |
| 898 | Milk Skimmed Dry | animal |
| 901 | Cheese of Whole Cow Milk | animal |
| 905 | Whey Cheese | animal |
| 907 | Processed Cheese | animal |
| 909 | Prod.of Nat.Milk Constit | animal |
| 976 | Sheep | animal |
| 977 | Sheep meat | animal |
| 978 | Offals of Sheep,Edible | animal |
| 984 | Cheese of Sheep Milk | animal |
| 1016 | Goats | animal |
| 1017 | Goat meat | animal |
| 1018 | Offals of Goats, Edible | animal |
| 1021 | Cheese of Goat Mlk | animal |
| 1034 | Pigs | animal |
| 1035 | Pig meat | animal |
| 1036 | Offals of Pigs, Edible | animal |
| 1037 | Fat of Pigs | animal |
| 1039 | Bacon and Ham | animal |
| 1041 | Sausages of Pig Meat | animal |
| 1042 | Prep of Pig Meat | animal |
| 1057 | Chickens | animal |
| 1062 | Hen eggs, in shell | animal |
| 1063 | Eggs Liquid | animal |
| 1064 | Eggs Dried | animal |
| 1069 | Duck meat | animal |
| 1073 | Goose and guinea fowl meat | animal |
| 1080 | Turkey meat | animal |
| 1091 | Other bird eggs,in shell | animal |
| 1096 | Horses | animal |
| 1097 | Horse meat | animal |
| 1107 | Asses | animal |
| 1110 | Mules | animal |
| 1159 | Offals other camelids | animal |
| 51 | Beer of Barley | luxury |
| 156 | Sugar cane | luxury |
| 157 | Sugar beet | luxury |
| 160 | Maple Sugar and Syrups | luxury |
| 161 | Sugar crops, nes | luxury |
| 162 | Sugar Raw Centrifugal | luxury |
| 164 | Sugar Refined | luxury |
| 165 | Molasses | luxury |
| 166 | Other Fructose and Syrup | luxury |
| 167 | Sugar, nes | luxury |
| 171 | Sugar flavoured | luxury |
| 172 | Glucose and Dextrose | luxury |
| 226 | Arecanuts | luxury |
| 278 | Oil of Jojoba | luxury |
| 563 | Must of Grapes | luxury |
| 564 | Wine | luxury |
| 565 | Vermouths&Similar | luxury |
| 566 | Marc of Grapes | luxury |
| 656 | Coffee, green | luxury |
| 657 | Coffee Roasted | luxury |
| 661 | Cocoa beans | luxury |
| 662 | Cocoa Paste | luxury |
| 664 | Cocoa Butter | luxury |
| 665 | Cocoapowder&Cake | luxury |
| 666 | Chocolate Prsnes | luxury |
| 667 | Tea | luxury |
| 677 | Hops | luxury |
| 687 | Pepper (Piper spp.) | luxury |
| 689 | Chillies and peppers, dry | luxury |
| 692 | Vanilla | luxury |
| 693 | Cinnamon (canella) | luxury |
| 698 | Cloves | luxury |
| 702 | Nutmeg, mace and cardamoms | luxury |
| 711 | Anise, badian, fennel, corian. | luxury |
| 720 | Ginger | luxury |
| 723 | Spices, nes | luxury |
| 826 | Tobacco, unmanufactured | luxury |
| 265 | Castor oil seed | other |
| 266 | Oil of Castor Beans | other |
| 269 | Sunflower Cake | other |
| 272 | Cake of Rapeseed | other |
| 273 | Olive Residues | other |
| 329 | Cottonseed | other |
| 332 | Cake of Cottonseed | other |
| 333 | Linseed | other |
| 334 | Linseed oil | other |
| 335 | Cake of Linseed | other |
| 541 | Stone fruit, nes | other |
| 663 | Cocoahusks;Shell | other |
| 767 | Cotton lint | other |
| 768 | Cotton Carded,Combed | other |
| 769 | Cotton Waste | other |
| 770 | Cotton Linter | other |
| 773 | Flax fibre and tow | other |
| 774 | Flax Tow Waste | other |
| 777 | Hemp Tow Waste | other |
| 780 | Jute | other |
| 782 | Other Bastfibres | other |
| 788 | Ramie | other |
| 789 | Sisal | other |
| 800 | Agave Fibres Nes | other |
| 809 | Manila Fibre (Abaca) | other |
| 821 | Fibre Crops Nes | other |
| 836 | Natural rubber | other |
| 919 | Cattle hides | other |
| 920 | Hides Wet Salted Cattle | other |
| 921 | Hidesdry S.Cattle | other |
| 998 | Skins Nes Sheep | other |
| 999 | Skins With Wool Sheep | other |
| 1025 | Goatskins | other |
| 1100 | Hair of Horses | other |
| 1103 | Hides Wet Salted Horses | other |
| 1104 | Hides Dry Slt Horses | other |
| 1105 | Hides Unsp Horse | other |
| 1187 | Cocoon Unr.&Waste | other |
| 1219 | Hair Coarse Nes | other |
